# Selection and Validation of Reference Genes for Real-Time Quantitative PCR in Hyperaccumulating Ecotype of *Sedum alfredii* under Different Heavy Metals Stresses

**DOI:** 10.1371/journal.pone.0082927

**Published:** 2013-12-10

**Authors:** Jian Sang, Xiaojiao Han, Mingying Liu, Guirong Qiao, Jing Jiang, Renying Zhuo

**Affiliations:** 1 State Key Laboratory of Tree Genetics and Breeding, Beijing, China; 2 Key Laboratory of Tree Genomics, The Research Institute of Subtropical Forestry, Chinese Academy of Forestry, Fuyang, Zhejiang, China; Universidade Federal do Rio Grande do Sul, Brazil

## Abstract

Real-time Quantitative PCR (RT-qPCR) has become an effective method for accurate analysis of gene expression in several biological systems as well as under different experimental conditions. Although with high sensitivity, specificity and broad dynamic range, this method requires suitable reference genes for transcript normalization in order to guarantee reproducible and meaningful results. In the present study, we evaluated five traditional housekeeping genes and five novel reference genes in Hyperaccumulating ecotype of *Sedum alfredii*, a well known hyperaccumulator for heavy metals phytoremediation, under Cd, Pb, Zn and Cu stresses of seven different durations. The expression stability of these ten candidates were determined with three programs - geNorm, NormFinder and BestKeeper. The results showed that all the selected reference genes except for *SAND* could be used for RT-qPCR normalization. Among them *UBC9* and *TUB* were ranked as the most stable candidates across all samples by three programs together. For the least stable reference genes, however, BestKeeper produced different results compared with geNorm and NormFinder. Meanwhile, the expression profiles of *PCS* under Cd, Pb, Zn and Cu stresses were assessed using *UBC9* and *TUB* respectively, and similar trends were obtained from the results of the two groups. The distinct expression patterns of *PCS* indicated that various strategies could be taken by plants in adaption to different heavy metals stresses. This study will provide appropriate reference genes for further gene expression quantification using RT-qPCR in Hyperaccumulator *S. alfredii*.

## Introduction

The contamination of soil and underground water by heavy metals is not only posing an unacceptable hazard to human and animal health but also placing an overwhelming burden on eco-environment [1,2]. In the past decade, exploiting natural hyperaccumulator plants to decontaminate heavy metal polluted soil, namely phytoremediation, has been regarded as an promising and eco-friendly strategy with low cost [3-5]. More than 400 plant species were already identified as natural heavy metal hyperaccumulators (account for about 0.2% of all angiosperms) [6], among them Hyperaccumulating ecotype of *S. alfredii* is a Cd/Zn/Pb co-hyperaccumulating species native to China which can also confer strong tolerance to heavy metals stresses [7]. To date, several critical physiological indices, such as accumulation capacity and translocation rate of different metal ions from roots to shoots and their regularities of distribution, have been characterized in this plant [8-11]. However, minimal knowledge is available on the molecular mechanism underlying hyper-accumulation and hyper-tolerance to heavy metals in *S. alfredii*. In order to explore the molecular mechanism and make full use of its gene resources for further phytoremediation breeding, it is necessary to dig out the candidate genes responsible for metal hyper-accumulation and analyze their expression patterns under heavy metals stresses [12,13].

Gene expression analysis by real-time quantitative PCR (RT-qPCR) is extensively used in many fields of plant biological research, including responses to abiotic and biotic stresses. Compared with conventional methods, RT-qPCR is more practical in gene expression analysis for its high sensitivity, specificity and a broad dynamic range even with limited amounts of RNA sample [14-16]. Regardless of its numerous advantages, this method requires appropriate normalization methods to ensure reliable and meaningful gene expression measurement. Among several strategies reported, it is the most popular to make use of suitable reference genes for gene expression normalization. The ideal reference genes should be expressed at a relatively constant level among samples from different tissues and developmental stages, and also should be unaffected by different experimental treatments [17-19]. During the previous gene expression analysis in *S. alfredii*, *ACT* was often used for RT-qPCR normalization [20-22], however, without any systematic study validating reference genes under abiotic stress reported for *S. alfredii*. Recent studies revealed that some traditional housekeeping genes such as *ACT*, *TUB* and *EF-1α* were not constantly stably expressed under different experimental conditions [23-25]. Therefore, it is necessary to perform a selection and validation of reference genes in a preliminary experiment to appraise their practicability for RT-qPCR normalization in *S. alfredii*.

In the present study, we compared the stability of ten candidate genes in Hyperaccumulating ecotype of *S. alfredii* exposed to four kinds of heavy metals: CdCl_2_, ZnSO_4_, Pb(NO_3_)_2_ and CuSO_4_ for seven different durations, respectively. Among them, five candidate genes (*ACT2*, *TUB*, *GADPH*, *UBC9*, *EF-1α*) are used as traditional housekeeping genes, while the others (*SAND*, *TIP41*, *AP-2*, *TBP*, *APT*) are relatively novel reference genes. Transcriptome data of Hyperaccumulating ecotype of *S. alfredii* was used for the source of potential reference genes. Our results will provide suitable RT-qPCR normalization for accurate gene expression analysis in Hyperaccumulating ecotype of *S. alfredii.*

## Results

### Identification of reference gene candidates and primer specificity and efficiency

**Figure 1:**
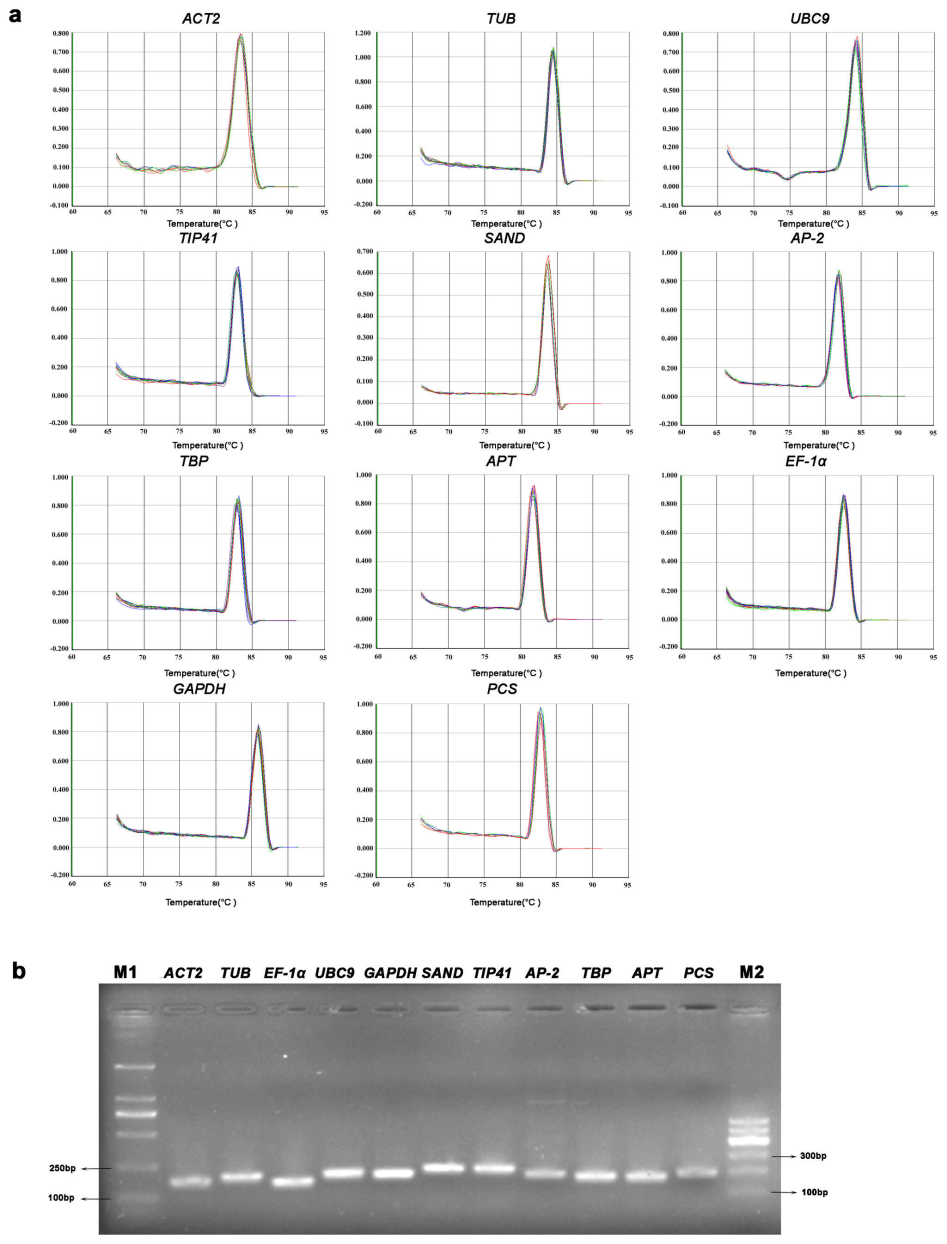
Confirmation of primer specificity and amplicon size. (a) Melting curves of ten candidate reference genes and one target gene showing single peaks. (b) Agarose gel (2%) showing specific RT-qPCR products of the expected size for each genes. M1 and M2 represent 2000bp and 500bp DNA marker respectively.

A total of ten reference genes were selected as potential candidates for RT-qPCR normalization through a search for the orthologs of *Arabidopsis* reference genes in Hyperaccumulating ecotype of *S. alfredii* transcriptome data. These genes are the orthologs of *AT3G18780* (Actin-2, *ACT2*), *AT1G20010* (beta-Tubulin, *TUB*), *AT4G27960* (Ubiquitin conjugating enzyme 9, *UBC9*), *AT4G34270* (TIP41-like family protein, *TIP41*), *AT2G28390* (SAND family protein, *SAND*), *AT5G46630* (AP-2 complex subunit mu-like, *AP-2*), *AT1G55520* (TATA binding protein, *TBP*), *AT1G27450* (Adenine Phosphoribosyltransferase, *APT*), *AT5G60390* (Elongation factor 1-alpha, *EF-1α*), *AT1G13440* (glyceraldehyde 3-phosphate dehydrogenase, *GAPDH*). To verify the accuracy of the selected gene sequences, RT-qPCR products were sequenced respectively, and their sequences were listed in the File S1. The estimated PCR amplification efficiency of these candidates varied from 1.963 for *GAPDH* to 2.082 for *TIP41*, and the correlation coefficients (*R* ^*2*^) ranged between 0.9964 for *AP-2* and 0.9997 for *APT*, respectively (Table 1). Melting curves analysis with single distinctive peak revealed that no primer dimers were generated from non-specific amplification (Figure 1a). Furthermore, the amplification specificity was confirmed by 2% agarose gel electrophoresis with single fragment of the expected size (Figure 1b). 

**Table 1:** Candidate reference genes, primers and different parameters derived from RT-qPCR analysis.

**Gene Symbol**	**GeneBank Accession nmuber**	**Primer sequence(forward/reverse)**	**Amplicon Length (bp)**	**Amplification efficiency**	**R^2^**
*ACT2*	KF652103	TTCCGGTGATGGTGTCAGTCA/ACAATTTCCCGCTCAGCAGTG	165	2.033	0.9980
*TUB*	KF652104	TTATGGCGATTCCGAGCTTCA/ATTATTTCCAGCGCCGGATTG	193	2.057	0.9981
*EF-1α*	KF652105	CATTATGAACCACCCCGGTCA/CCGCATCTCCATTCTTCAGGA	164	2.071	0.9979
*UBC9*	KF652106	TGGCGTCGAAAAGGATTCTGA/CCTTCGGTGGCTTGAATGGATA	198	2.083	0.9969
*GAPDH*	KF652107	AAGAGCCGCCTCGTTCAACAT/TGATTGCCGCCTTGATCTCAT	191	1.975	0.9965
*SAND*	KF652108	TGGAGCGAAACCCTAGCTTCA/TCATCCACGCGGATTGAACTT	211	2.073	0.9980
*TIP41*	KF652109	ATGCACTTGCTGGATGGAAGC/TCCTTGCAGTCCTCCCATTGA	223	1.963	0.9996
*AP-2*	KF652110	ACTTTGGTGGGGCTTTTGACG/GCAAGGTTGCATTGGGAACAG	195	2.027	0.9964
*TBP*	KF652111	ATGGCTGAGCAAAGCATGGAG/CACAGCAGCAAAACGCTTAGGA	180	2.024	0.9967
*APT*	KF652112	GCGCTAGCAATTGGTGCAAAA/GCAGCACATAAAGTGCCACCA	191	2.059	0.9997
*PCS*	KF652113	TGTTGTGGCTCCAACCCTTG/TTGACGCAAGTGCATCACCTC	192	1.915	0.9990

### Expression levels of the selected candidate reference genes

**Figure 2:**
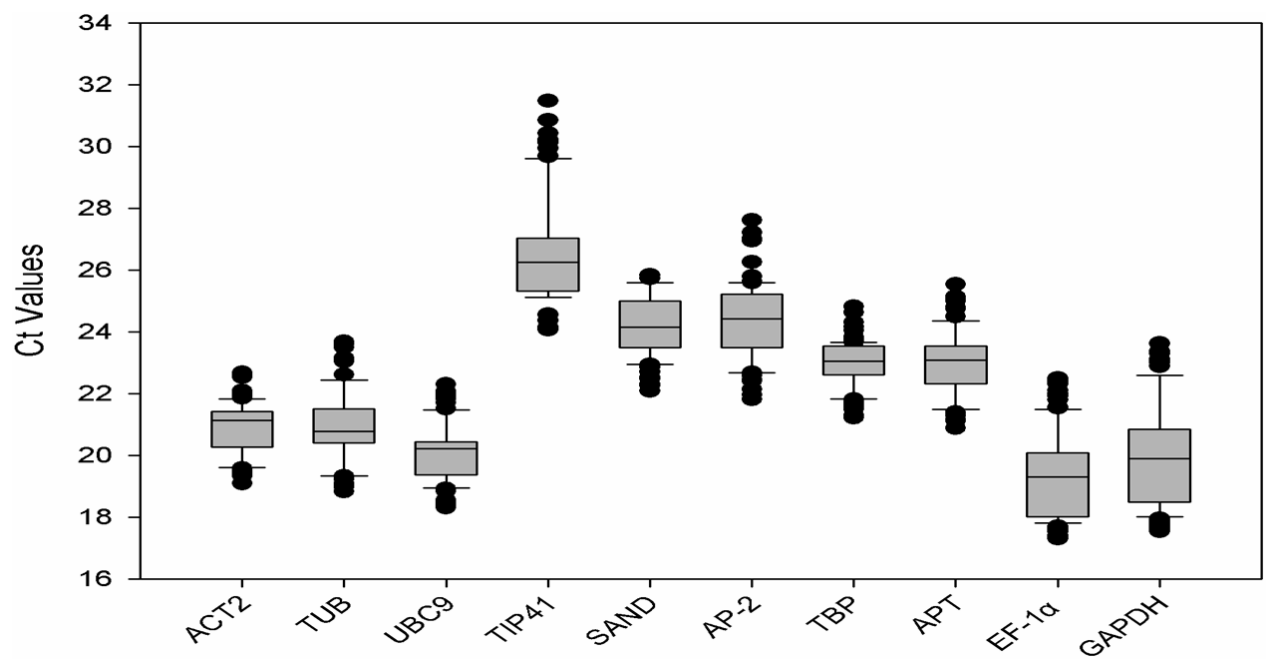
RT-qPCR Ct values for reference genes. Ct values for each reference gene in all Hyperaccumulating ecotype of *S. alfredii* samples. A line across the box depicts the median. The box indicates the 25% and 75% percentiles, whiskers caps represent the maximum and minimum values, dots represent outliers.

The average cycle threshold (Ct) values were calculated to reveal the differences in transcript levels among the reference genes with different heavy metals treatments. The ten candidates showed a relatively wide dispersal of transcript levels with average Ct values ranged from 19.29 for *EF-1α* to 26.26 for *TIP41* (Figure 2), and most of them were moderately abundant (lying between 20 and 25). Among them *EF-1α* was the most abundant reference gene and *TIP41* was the least one. The coefficient of variance (CV) values were also computed to reveal the expression stability of these reference genes. *TBP* had the least variation with a CV value of 2.32%, while *GAPDH* was the most variable one with a CV value of 6.53%. 

### Expression stability of candidate reference genes

Three different programs were applied to evaluate the expression stability of the selected reference gene candidates: geNorm [26], NormFinder [27] and BestKeeper [28]. To select stably expressed genes, we collected Ct values data across all samples. These Ct data were used directly for stability calculations for BestKeeper analysis, and then transformed to relative quantities using the delta-Ct method for geNorm and NormFinder analysis.

#### a): geNorm analysis

**Figure 3:**
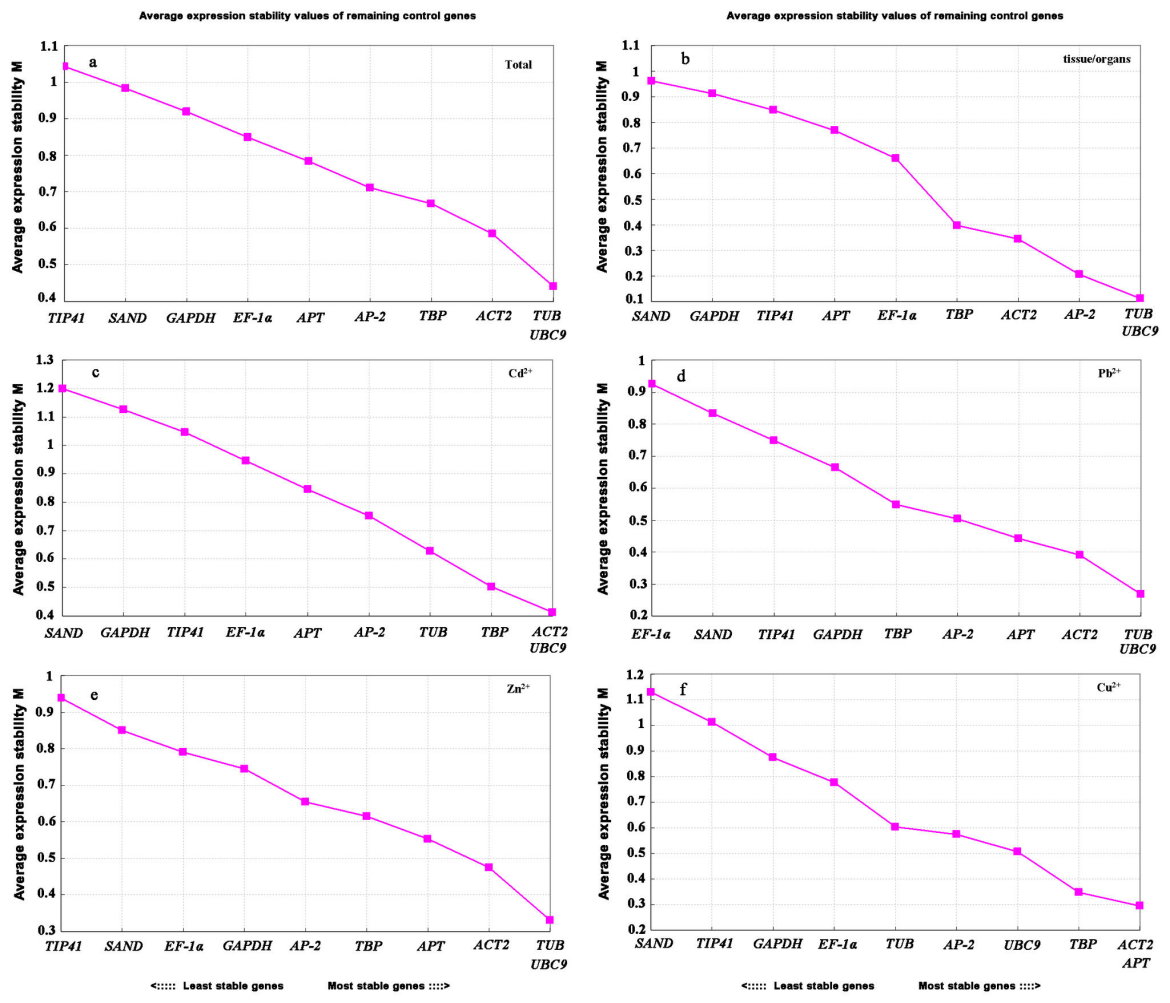
Expression stability (*M*) of ten reference genes across all 31 samples calculated by geNorm. (a) all 31 samples, (b) different tissue/organs, (c) Cd^2+^ treatment, (d) Pb^2+^ treatment, (e) Zn^2+^ treatment, (f) Cu^2+^ treatment.

The expression stability measure *M* for all selected reference genes was calculated by geNorm program based on the average pairwise variation between all the tested reference genes. The most stable reference gene has the lowest *M* value, whereas the least stable one has the highest *M* value [26]. In our analysis, all reference genes under different heavy metals stresses had an *M* value less than the geNorm threshold of 1.5 that could be recognized as stable (Figure 3). For treatments with Zn and Pb, *UBC9* and *TUB* were the most stably expressed reference genes, which was consistent with the results from different plant organs. *ACT2* and *UBC9* were ranked as the most stable under Cd stress, while *ACT2* and *APT* were ranked as the most stable under Cu stress. On the whole, *UBC9* and *TUB* had the lowest *M* value with the most stability and *TIP41* had the least stability across all samples. The discrepancy existed in stability ranking of reference genes under different heavy metals stresses revealed that virous strategies were taken by Hyperaccumulating ecotype of *S. alfredii* in response to specific heavy metal.

**Figure 4:**
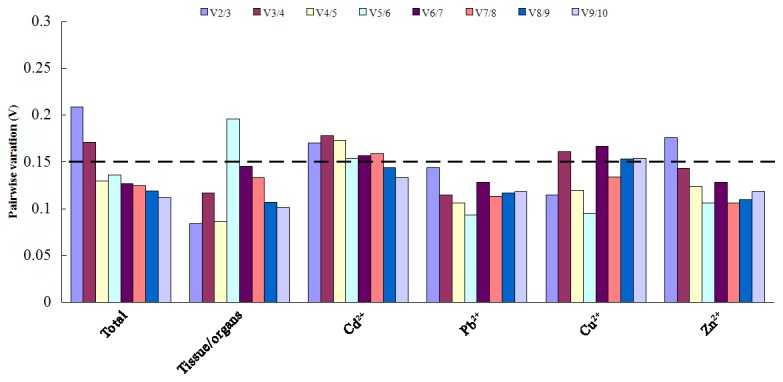
Pairwise variation (V) calculated by geNorm to determine the optimal number of reference genes. The average pairwise variations (Vn/Vn+1) were analyzed to measure the effect of adding additional reference gene on the RT-qPCR normalization for all samples.

To determine the optimal number of reference genes required for reliable normalization, geNorm was used to calculate their pairwise variation (V_n_/V_n+1_) between two sequential normalization factors NF_n_ and NF_n+1_ (Figure 4). Analysis of the pairwise variation for all samples showed that the V_2/3_ value (0.209) and V_3/4_ (0.171) were higher than the recommended threshold of 0.15, which was universally accepted as the cutoff value [25]. When the fourth reference gene was included, V_4/5_ value (0.130) dropped below 0.15, indicating that four reference genes would be sufficient for proper gene expression normalization in all samples of Hyperaccumulating ecotype of *S. alfredii.*

#### b): NormFinder analysis

Another adopted program NormFinder is used to calculate the stability value of reference genes based on their intra- and inter-expression variation of expression [27]. Those exhibited lower average expression stability values are regarded as more stably expressed reference genes. The stability values for all the reference genes by NormFinder calculation were shown in Table 2. Although the stability ranking of the ten candidates with this method showed somewhat different from geNorm under specific heavy metals stresses, when considering all samples together, the five most stable reference genes: *UBC9* (0.304), *TUB* (0.319), *ACT2* (0.322), *TBP* (0.395) and *AP-2* (0.459) were ranked consistently with geNorm. *SAND* and *TIP41* were identified as the least stable candidates by both geNorm and NormFinder. Similar to geNorm program, NormFinder also displayed a difference in the stability ranking of reference genes with different heavy metal treatments.

**Table 2:** Ranking of candidate reference genes in order of their expression stability as calculated by NormFinder.

**Rank**	**Total**	**Tissue/organs**	**Cd**	**Pb**	**Zn**	**Cu**
1	*UBC9* (0.304)	*TBP* (0.094)	*UBC9* (0.143)	*UBC9* (0.093)	*TUB* (0.110)	*TUB* (0.207)
2	*TUB* (0.319)	*ACT2* (0.304)	*TUB* (0.178)	*ACT2* (0.161)	*APT* (0.220)	*APT* (0.268)
3	*ACT2* (0.322)	*TUB* (0.438)	*ACT2* (0.415)	*TUB* (0.188)	*UBC9* (0.297)	*ACT2* (0.274)
4	*TBP* (0.395)	*AP-2* (0.438)	*TBP* (0.514)	*TBP* (0.338)	*TBP* (0.37)	*AP-2* (0.315)
5	*AP-2* (0.459)	*UBC9* (0.517)	*EF-1α* (0.551)	*APT* (0.350)	*AP-2* (0.438)	*UBC9* (0.327)
6	*EF-1α* (0.518)	*APT* (0.563)	*APT* (0.619)	*AP-2* (0.356)	*ACT2* (0.481)	*TBP* (0.432)
7	*APT* (0.559)	*EF-1α* (0.570)	*AP-2* (0.782)	*TIP41* (0.602)	*GAPDH* (0.523)	*EF-1α* (0.694)
8	*GAPDH* (0.659)	*GAPDH* (0.605)	*TIP41* (0.793)	*GAPDH* (0.621)	*EF-1α* (0.549)	*GAPDH* (0.742)
9	*TIP41* (0.759)	*SAND* (0.679)	*SAND* (0.897)	*SAND* (0.748)	*SAND* (0.711)	*TIP41* (1.002)
10	*SAND* (0.761)	*TIP41* (0.734)	*GAPDH* (0.914)	*EF-1α* (0.806)	*TIP41* (0.806)	*SAND* (1.054)

#### c): BestKeeper analysis

BestKeeper program is used to evaluate the expression stability of reference genes based on their coefficient of correlation (r) to the BestKeeper Index (BI) by the calculation of standard deviation (SD) and percentage covariance (CV) [28]. In the present study, the BestKeeper analysis revealed that the best correlations were obtained for *TUB* (0.945) and *UBC* (0.878) with *P* value of 0.001 across all samples, while *SAND* (0.247) with a P value as high as 0.181 was excluded (Table 3). However, the two unstable reference genes *GAPDH* and *TIP41* identified by geNorm and NormFinder got relatively high correlation coefficient value of 0.878 and 0.866, which might result from the distinct adopted statistical algorithms. Therefore *UBC9* and *TUB* were ranked as the most stable reference genes and *SAND* was ranked as the least one by three programs together in Hyperaccumulating ecotype of *S. alfredii* under different heavy metals stresses.

**Table 3:** Statistics results by BestKeeper program for ten selected genes based on Ct values.

**Rank**	**Gene name**	***r***	***P*-value**	**CV(%)**	**SD**
1	*TUB*	0.95	0.001	3.95	1.8
2	*UBC9*	0.88	0.001	3.29	1.6
3	*GAPDH*	0.87	0.001	6.53	2.52
4	*TIP41*	0.83	0.001	4.49	2.33
5	*ACT2*	0.80	0.001	3.03	1.57
6	*EF-1α*	0.79	0.001	5.27	2.07
7	*AP-2*	0.79	0.001	3.77	1.92
8	*TBP*	0.72	0.001	2.32	1.46
9	*APT*	0.67	0.001	3.30	1.71
10	*SAND*	0.25	0.181	3.27	1.75

### Validation of the selected reference gene candidates

**Figure 5:**
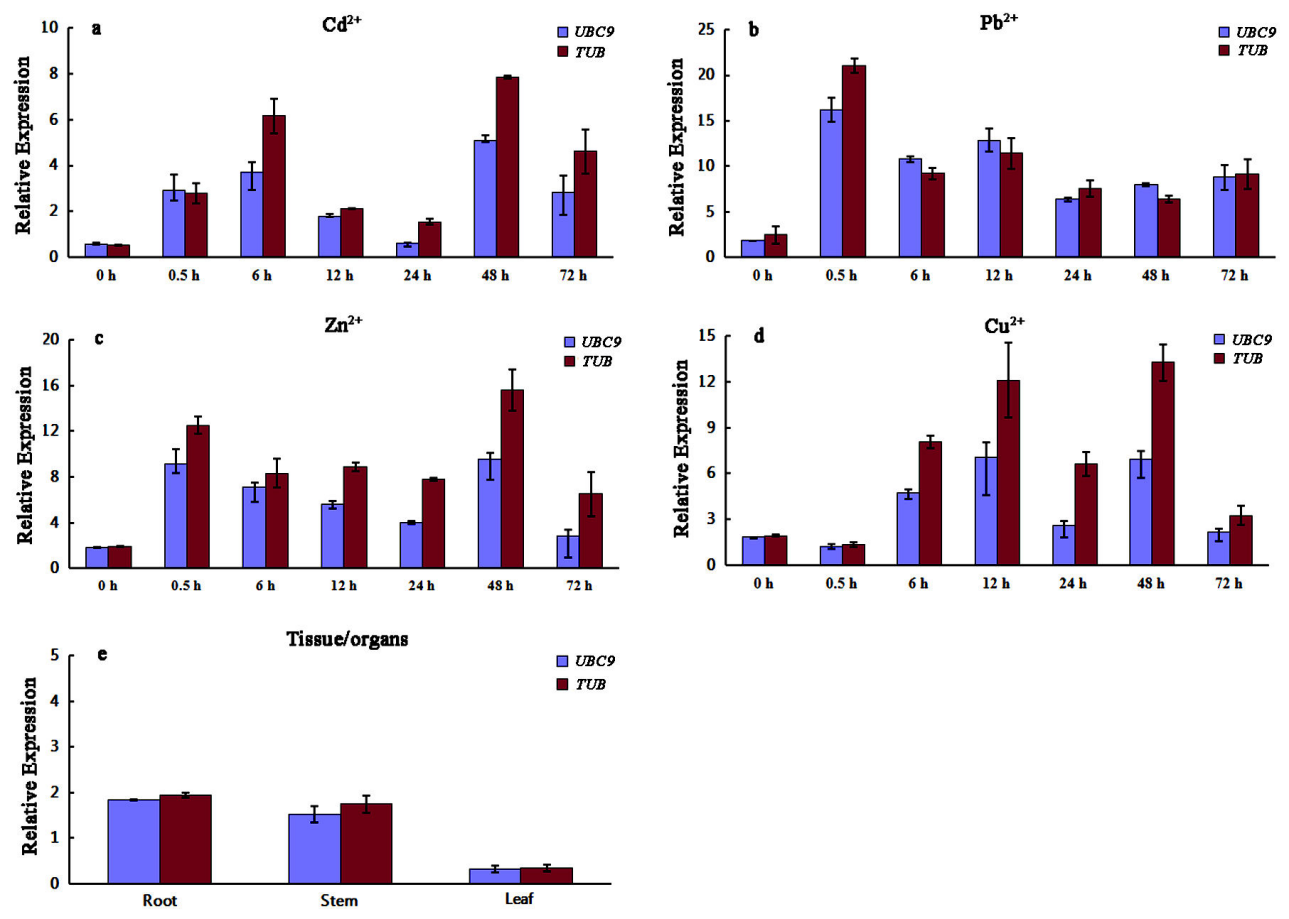
The expression quantification of *PCS* using UBC9 and *TUB* as internal controls under different heavy metals stresses for seven durations and in different tissue/organs. (a) Cd^2+^ treatment, (b) Pb^2+^ treatment, (d) Zn^2+^ treatment, (d) Cu^2+^ treatment, (e) different tissue/organs without any heavy metals treatments.

In order to ensure the reliability of the selected reference gene candidates, we analyzed the expression patterns of phytochelatin synthase (PCS) across all samples under different heavy metals stresses. The two most appropriate reference genes *UBC9* and *TUB* selected by geNorm, NormFinder and BestKeeper were used for the RT-qPCR normalization of *PCS*, respectively. Phytochelatins, produced by the enzyme phytochelatin synthase (PCS), have been reported to be a class of cellular chelating agents which can play an important role in heavy metal detoxification, and it can be activated by various heavy metal ions [5]. The normalization of RT-qPCR using *UBC9* and *TUB* generated similar expression patterns of *PCS* (Figure 5). An increased transcript level of *PCS* induced by heavy metals could be detected, while the specific expression patterns of *PCS* under Cd, Pb, Zn and Cu stresses were different from each other. The relative expression of *PCS* in different organs without any heavy metals treatments was also calculated. Its transcript was expressed relatively higher in roots and stems than leaves, and no significant difference was found when using the two different reference genes for normalization, respectively. 

## Discussion

Nowadays, RT-qPCR has become an effective method for gene expression patterns analysis in plant biological studies. Since the relative expression of target genes can be influenced significantly by reference genes, it is necessary to select ideal reference genes for the reliable and reproducible gene expression measurement. Some commonly used traditional housekeeping genes were doubted in recent studies for their variable expressions when tested with different experimental treatments [23]. The application of novel genes then became more and more popular for gene expression analysis with RT-qPCR [33-36]. However, our results showed that traditional housekeeping genes were expressed more stable than novel genes for RT-qPCR normalization under different heavy metals stresses in *S. alfredii*. Thus, the selection of reference genes across all samples under different experimental conditions is indeed an essential job before further gene expression analysis.

As a Cd/Zn/Pb co-hyperaccumulator, *S. alfredii* is well known for its potential utilization in the detoxification of contaminated soils by heavy metals [7-11]. Previous studies of the mechanism involved in heavy metals hyper-accumulation and hyper-tolerance for this plant were generally focused on physiological view, which was unable to reveal its intrinsic characteristic essentially. So far, no large-scale related molecular biological research has been carried out in *S. alfredii* due to limited available sequence information. Taking advantage of transcriptome data, a precious sequence resource for non-model plant systems, many heavy metals stresses-responsive genes will be identified with comprehensive functional analysis in the near future. In this study, we performed a selection and validation of reference genes for further accurate gene expression analysis using RT-qPCR in *S. alfredii*.

Three Excel-based programs geNorm, NormFinder and BestKeeper were used in the present study to evaluate the expression stability of ten selected reference gene candidates in the total of 31 samples. Those samples were extracted from Hyperaccumulator *S. alfredii* with four kinds of heavy metal treatments (CdCl_2_, Pb(NO_3_)_2_, CuSO_4_ and ZnSO_4_) for seven process durations: 0 h, 0.5 h, 6 h, 12 h, 24 h, 48 h and 72 h, respectively. Although with different statistical algorithms and analytical methods, same results were produced by three programs that *UBC9* and *TUB* were the most stably expressed reference genes and *SAND* the least one across all samples. However, as the unstable candidates selected by both geNorm and NormFinder, *GAPDH* and *TIP41* were ranked relatively stable by BestKeeper. This inconsistent result indicated that using geNorm and NormFinder in some cases might not be enough for the studies on reference genes selection. Although some previous studies only used the two programs to select reference genes successfully [29-32], we suggested that taking into consideration the stability ranking of BestKeeper could make the results more convincing. Besides the different stability ranking resulted from distinct algorithms, another discrepancy was observed in the stability ranking with four kinds of heavy metals treatments using same software, which might imply that diverse reactions appeared in Hyperaccumulator *S. alfredii* in response to different heavy metals stresses. In previous studies, *ACT* was often used as internal control for expression quantification of heavy metals stresses responsive genes in *S. alfredii* [20-22]. In our study, *ACT* was also regarded as stable reference genes, however, *UBC9* and *TUB* were suggested to be better for RT-qPCR normalization in *S. alfredii*, which might provide more accurate expression measurement of target genes.

To validate the reference gene candidates selected by three programs, the transcript level of phytochelatin synthetase (PCS) under different heavy metals stresses were quantified using the two most stable reference genes *UBC9* and *TUB* for RT-qPCR normalization, respectively. There was no obvious difference existed in the expressional trends of *PCS* generated by normalization of *UBC9* compared with that by normalization of *TUB* for all samples, further indicating that both *TUB* and *UBC9* were appropraite reference genes. A heated debate occurred several years ago on whether phytochelatin synthetase could be induced by heavy metals in hyperaccumulator *S. alfredii*. As reported by Sun et al., phytochelatin (PC) could not be detected using the physiological methods - HPLC under Cd, Pb and Zn stresses [36]. While Zhang et al. reported that PC formation could be induced by Cd and Pb, but not Zn stress with the same HPLC method [37]. Our expression analysis of *PCS* using RT-qPCR indicated that *PCS* could be induced by Cd, Pb, Zn and Cu treatments at transcript levels with different expression patterns in hyperaccumulator *S. alfredii*. This discrepancy might be resulted from distinct expression regulations of *PCS* between transcription and translation levels under different heavy metals stresses, which is worth for further exploration.

## Conclusion

To sum up, five traditional housekeeping genes and five novel reference genes from Hyperaccumulator *S. alfredii* were evaluated under four kinds of different heavy metals stresses and in different organs using geNorm, NormFinder and BestKeeper programs. The results showed that *UBC9* and *TUB* were the most appropriate reference genes for the RT-qPCR normalization across all samples. Furthermore, we validated the two optimal reference genes in all samples by quantifying the expression level of phytochelatin synthetase. This study constitutes the first systematic exploration in *S. alfredii* to identify optimal reference genes for RT-qPCR normalization under different heavy metals stresses.

## Materials and Methods

### Ethics Statement

These field studies did not involve any protected species. No specific permits were required for the collection of samples in the study location.

### Plant materials and treatments

 Hyperaccumulating ecotype of *S. alfredii* were collected from an old Pb/Zn mined area in Quzhou city, Zhejiang Province, PR China. After 14 d water-cultivation in artificial climate chest at 25~28°C under long day (16 hours light/8 hours dark) conditions, the plants were treated with 400 μM CdCl_2_, 400 μM Pb(NO_3_)_2_, 500 μM CuSO_4_ and 500 μM ZnSO_4_, respectively. Then the roots of the treated plants were sampled at 0 h, 0.5 h, 6 h, 12 h, 24 h, 48 h and 72 h. Three different tissue/organs including roots, stems and leaves of the plants without any heavy metals treatments were also sampled. Each treatment was replicated three times. All the samples were frozen in liquid nitrogen immediately after collection and then stored at - 80°C for further study. 

### Total RNA extraction and first strand cDNA synthesis

Total RNA was extracted using the Total RNA Purification Kit (NORGEN, Thorold, Canada) and then treated with DNase I (TaKaRa Bio, Dalian, China) to remove genomic DNA contamination according to the manufacturer’s recommendations. The integrity of total RNA was checked with denaturing 1.0% (p/v) agarose gel electrophoresis. A NanoDrop2000 spectrophotometer (Thermo, Wilmington, USA) was then used to determine the purity and concentration of the RNA samples. Only those with appropriate absorbance ratio of A260/A280 and A260/A230 were used for cDNA synthesis. After that, 1 μg of RNA sample was used for the first strand cDNA synthesis in a total volume of 20 μL with the superscript Ш first strand synthesis system followed by the RNase H step (Invitrogen, Carlsbad, USA). The cDNA　products were diluted 30-fold prior to use in RT-qPCR.

### PCR primer design

The sequences of ten reference gene candidates and one target gene were obtained from Hyperaccumulating ecotype of *S. alfredii* transcriptome data by BLAST search using the sequences of reported Arabidopsis reference genes with score value higher than 250 and identity value higher than 95%. Primer3 program (http://frodo.wi.mit. edu/primer3/) was used to design the primers pairs for all those genes with the optimal Tm at 58-60°C, product size ranging from 150 to 200 bp, primer length between 19 and 21 nucleotides and a GC content of 45 to 55%. The products of real-time PCR with a 2% agarose gel electrophoresis and melt curve analysis were conducted to check the specificity of all primers. 

### RT-qPCR

RT-qPCR amplification was performed in 96-well plates using a 7300 Real Time PCR System (Applied Biosystems, CA, USA) with SYBR® Premix Ex Taq^TM^ Kit (TaKaRa Bio, Dalian, China) according to the manufacturer’s protocol. Each PCR reaction was performed in 20 μl volumes containing: 2 μl cDNA, 10 μl SYBR® Premix Ex Taq^TM^, 0.4 μl of both 10 μM forward primer and reverse primer, 6.8 μl PCR-grade water and 0.4 μl ROX reference dye. The standard thermal profile used in RT-qPCR was as follows: 95°C for 10 s，40 cycles of 95°C for 5 s and 60°C for 31 s. After that, a dissociation curve was generated (from 60°C to 95°C) to check the specificity of amplicons. Threshold cycles (Ct) were automatically determined and each PCR reaction was performed in triplicate.

### Analysis of gene stability

Standard curves were used to calculate the gene-specific PCR efficiency from 5-fold series dilution of the mixed cDNA template for each primer pair. The correlation coefficients (*R* ^*2*^) and slope values were acquired from the standard curve, and PCR amplification efficiencies (*E*) were calculated according to the equation *E* = (5^-1/slope^ - 1) ×100%.

Three Excel-based softwares geNorm, NormFinder and BestKeeper were used to evaluate the expression stability of all the candidate genes. The geNorm software is used to calculate the expression stability value (M) for reference genes based on the mean pairwise variation between all the tested genes (http://medgen.ugent.be/~jvdesomp/genorm/), and NormFinder is used to rank the expression stability of reference genes according to their intra- and interexpression variation of expression (http://www.mdl.dk/publicationsnormfinder.htm). While BestKeeper uses an index for the evaluation of expression stability which is calculated by standard deviation (SD) and percentage covariance (CV) (http://www.gene-quantification.de/bestkeeper.html).

## Supporting Information

File S1 **Sequence of the RT-qPCR products for the eleven selected genes (Their primers sequences were highlighted).**
(DOC)
