# Supplementary material for: Selection and Validation of Reference Genes for Real-Time Quantitative PCR in Hyperaccumulating Ecotype of Sedum alfredii under Different Heavy Metals Stresses
Source: PLoS One. 2013 Dec 10;8(12):e82927. doi: 10.1371/journal.pone.0082927 (PMC3858333; doi:10.1371/journal.pone.0082927)
Supplement: File S1 — Sequence of the RT-qPCR products for the eleven selected genes (Their primers sequences were highlighted). (DOC) [file pone.0082927.s001.doc]

**File S1 Sequence of the RT-qPCR products for the eleven selected genes (Their primer sequences were highlighted) .**

**>ACT2**

TTCCGGTGATGGTGTCAGTCACACAGTGCCCATTTATGAAGGTTATGCC**T**TGCCCCATGCCATCCTCCGTCTTGATCTTGCTGGACGTGATTTGACTGATTCTTTGATGAAAATTCTTACTGAAAGAGGTTACATGTTCACAACCACTGCTGAGCGGGAAATTGT

**>TUB**

TTATGGCGATTCCGAGCTTCAGCTTGAGAGGGTCAACGTGTATTACAATGAGGCTAGCGGTGGAAGGTTTGTTCCCAGAGCGGTGTTGATGGATCTGGAGCCTGGGACTATGGACAGCATTAGATCTGGACCTTATGGCAAGATCTTTAGGCCTGATAACTTTGTCTTTGGACAATCCGGCGCTGGAAATAAT

**>UBC9**

TGGCGTCGAAAAGGATTCTGAAAGAGCT**T**AAGGATCTTCAGAAGGATCCTCCTACTTC**T**TGCAGCGCTGGGCCTGTGGC**A**GAGGATATGTTTCACTGGCAAGCAACAATAATGGGTCCTGCTGATAGTCCATATGCCGGTGGAGTGTTTCTTGTTACCATTCATTTTCCTCCGGATTATCCATTCAAGCCACCGAAGG

**>SAND**

TGGAGCGAAACCCTAGCTTCAAGAGTGCTGACTGTGGAGTTCAAGAGGATCGATTGACGTGTTTGAGATTGGGTGGTGAGATCGATGTCGATGAATCTGGGTCTGATAAGGTCCCGGTGAAGGATGATCGTTGTTGCATTTCTGTCATTGAGAAGGATGAGCAGCATGATGAGGAGGAGTTGGAAGGGGGAAGTTCAATCCGCGTGGATGA

**>AP-2**

ACTTTGGTGGGGCTTTTGACGAGGATGCTATCCGGAACAATT**C**TGTTCTGATCTATGAGTTGCTGGATGAAATAATGGATTTTGGATATCCTCAAAATCTGTC**A**CCTGAGATTTTGAAGTTATATATCACGCAAGAGGGTGTGCGATCTCATCTGATGTCCAAGCCTGAGAAACCTGTTCCCAATGCAACCTTGC

**>TBP**

ATGGCTGAGCAAAGCATGGAGGGGAGTGAGCCAATCGATCCCATCAAGCATCCGTCTGGAATTGTACCTACTCTTCAGAACATTGTTTCCACTGTGAATCTTGACTGCAAGTTAGACTTGAAATCTATTGCTCTGCAGGCTCGTAATGCGGAGTACAATCCTAAGCGTTTTGCTGCTGTG

**>TIP41**

ATGCACTTGCTGGATGGAAGCAGGAGGCACTCCCTCCTGTTGAGGTTCCTGCTGCTGCTAAGTGGAAATTCAGGAGTAAACCCCTCAAGCAAGTCATATTAGATTACGATTACACATTTACAACACCATATTGTGGAAGTGAAACAATGGAGGTAGCTTCAAAGCAGGGGGTGAGAGAAATATCTGATGGGAGCTGCAGTCTTCAATGGGAGGACTGCAAGGA

**>APT**

GCGCTAGCAATTGGTGCAAAATTCGTTCCCTTGAGAAAACCCCGTAAATTGCCTGGACCTGTTATTTCTGAAGAGTATTCATTAGAGTACGGAACTGATAAAATAGAGATGCACATTGGAGCTGTACATGAAGGAGAACGTGTCCTGATTATCGACGATTTGATTGCTACTGGTGGCACTTTATGTGCTGC

**>EF-1α**

CATTATGAACCACCCCGGTCAGATTGGTAATGGTTATGCCCCAGTTTTGGATTGTCACACATCGCACATTGCTGTTAAGTTTGCTGAGATCCTAACCAAGATTGACAGACGGTCTGGTAAAGAACTCGAGAAGGAACCTAAGTTCCTGAAGAATGGAGATGCGG

**>GAPDH**

AAGAGCCGCCTCGTTCAACATCATCCCCAGCTCTACTGGAGCTGCTAAGGCTGTTGGGAAGGTGCTACCCGCGCTAAACGGAAAGCTTACTGGAATGGCCTTCCGCGTCCCAACTGTTGACGTCTCAGTTGTTGACCTTACTGTCAGGCTAGAGAAGGCTGCCACTTATGATGAGATCAAGGCGGCAATCA

**>PCS**

TGTTGTGGCTCCAACCCTTGCACATGTACCGGACCGACTGCTGGTGATGTTTTAACTGTACTTTTACTCTCTCTGCCTCCGCGTACATGGTCTGGTATAAAAGATGAAAAACTTTTGCATGAGATGCATAACCTCGTTTCAATCGACAACCTTCCACCTCTGCTTCAAGAAGAGGTGATGCACTTGCGTCAA
